# Vimentin Fragmentation and Its Role in Amyloid-Beta Plaque Deposition in Alzheimer’s Disease

**DOI:** 10.3390/ijms26072857

**Published:** 2025-03-21

**Authors:** Lan Zhang, Ji Wang, Yalong Yan, Lihong Xiang, Xinyue Zhai, Lianmei Cai, Zhuoran Sun, Mingshan Pi, Qi Xiong, Hongyan Zhou, Yuran Gui, Xiaochuan Wang, Xiji Shu, Yiyuan Xia

**Affiliations:** 1 Hubei Key Laboratory of Cognitive and Affective Disorders, School of Medicine, Jianghan University, Wuhan 430056, China; 2 Institutes of Biomedical Sciences, School of Medicine, Jianghan University, Wuhan 430056, China; 3 Department of Pathology and Pathophysiology, School of Medicine, Jianghan University, Wuhan 430056, China

**Keywords:** vimentin, Alzheimer’s disease, AEP, Aβ, ApoE

## Abstract

Intermediate filament protein vimentin (Vim) is a well-established marker for reactive astrocytes and has been closely associated with Alzheimer’s disease (AD). RNA sequencing data reveal elevated expression of Vim in AD brains, with its aggregation frequently observed around amyloid-β (Aβ) plaques. However, the precise mechanisms by which Vim influences the aggregation or propagation of Aβ plaques remain unclear. In this study, we detected the upregulation of astrocytic Vim in AD brain tissue, with its co-localization around Aβ plaques. Asparagine endopeptidase (AEP), another molecule implicated in AD, was found to cleave Vim both in vitro and in vivo, including within human brain tissue. Mass spectrometry analysis confirmed that the AEP cleavage site on Vim is located at N283. We further investigated the in vivo cellular localization of Vim and observed that fragmented Vim, particularly the C-terminal fragment Vim 284–466, promotes apoptosis and disrupts the network structure that is essential for interaction with glial fibrillary acidic protein (GFAP). This disruption impairs astrocytic phagocytosis of exogenous Aβ, which is attributed to the reduced release of apolipoprotein E (ApoE) by astrocytes. The decrease in ApoE levels, in turn, diminishes the transport and clearance of Aβ. Conversely, mutation of the Vim N283 site (N283A) prevents AEP-mediated cleavage of Vim, preserves the GFAP network structure, restores ApoE levels, and reverses the effects on Aβ aggregation. Collectively, our findings elucidate the role of Vim fragmentation in Aβ plaque deposition and propose a potentially novel therapeutic strategy for Alzheimer’s disease.

## 1. Introduction

Alzheimer’s disease (AD) is a neurodegenerative disorder marked by a relentless decline in cognitive function, memory loss, diminished linguistic capabilities, and impaired judgment [1]. It represents the predominant cause of age-related dementia worldwide. With the global population aged ≥ 65 years projected to reach 1.5 billion by 2050 [2], the escalating prevalence of AD underscores an urgent need to elucidate its molecular pathogenesis. While the classical neuropathological triad of amyloid-β (Aβ) plaques, neurofibrillary tau tangles, and synaptic loss remains central to AD diagnosis [3]. The etiological mechanisms linking these hallmarks to clinical manifestations remain incompletely understood. Notably, mutations that cause early-onset Alzheimer’s disease (EOAD) strongly support the notion that inhibiting Aβ aggregation will prevent the disease [4]. These genetic studies highlight Aβ as a central factor in the neurodegenerative cascade. In addition, the “amyloid hypothesis” proposes a serial model of causality, where elevated Aβ drives other disease features, including tau hyperphosphorylation [5]. Thus, the presence of Aβ plaques is considered a critical factor contributing to neuronal damage and dysfunction [6].

Vimentin (Vim), a key component of the cytoskeleton in most eukaryotic cells [7], morphologically presents intermediate filaments that are nearly identical across various cell types, including epithelial cells, muscle cells, and neurons. The distinctive structural attributes of Vim were first uncovered in 1982 through gene cloning and sequencing in hamsters, with subsequent research revealing a broad spectrum of homology sequences from fish to humans, establishing Vim as a highly conserved protein [8]. Studies indicate that Vim knockout (Vim^−/−^) mice display no overt phenotype in development and reproduction, suggesting that Vim, while a cytoskeletal protein, is not indispensable [9]. Nevertheless, Vim is acknowledged for its pivotal role in modulating cellular mechanics, encompassing the coordination of mechanosensing, signal transduction, motility, and inflammatory responses [10]. In neurodegenerative diseases, Vim has been implicated in the regulation of neuroplasticity and scar formation after nerve injury [11]. For instance, the upregulation of Vim and GFAP in astrocytes is indicative of activated and reactive glial hyperplasia in the face of injury, ischemia, or neurodegeneration [12]. Vim^−/−^ mice exhibit impaired post-traumatic synaptic plasticity and hippocampal neurogenesis, adversely affecting learning and memory functions [13,14,15]. Mechanistically, GFAP becomes reactive in AD in response to Aβ plaques [16], and double knockout mice for GFAP and Vim display a reduced interaction of astrocyte processes with plaques [17]. Spatially, GFAP/Vim appears more intimately associated with Aβ. However, the precise mechanisms by which Vim regulates reactive astrocytes and its detailed effects on Aβ plaques remain undefined.

Recent advances implicate cysteine proteases in modulating cytoskeletal dynamics during neurodegeneration. Asparagine endopeptidase (AEP/δ-secretase), a cysteine protease aberrantly activated in AD, specifically cleaves peptide bonds on the C-terminal side of asparagine residues [18]. Our group previously demonstrated that AEP-mediated proteolysis generates neurotoxic APP and tau fragments that synergistically accelerate Aβ/tau pathology [19,20]. Building upon these findings, we hypothesize that AEP may coordinate cytoskeletal destabilization through the cleavage of structural proteins like Vim.

In this study, we identify Vim as a novel AEP substrate and characterize its pathological cleavage into N-terminal (1–283) and C-terminal (284–466) fragments. Through in vitro and in vivo models, we demonstrate that AEP mediates Vim proteolysis. The Vim fragments generated by AEP increase cell mortality and impair the phagocytic function of astrocytes toward Aβ plaques by decrease ApoE level, leading to an increase in extracellular Aβ aggregation, and induces synaptic toxicity reversible through AEP inhibition. These results establish Vim fragmentation as a previously unrecognized mechanism linking protease dysregulation to AD progression.

## 2. Results

### 2.1. AEP and Vim Escalate and Interact with Each Other in AD Brain

**Figure 1:**
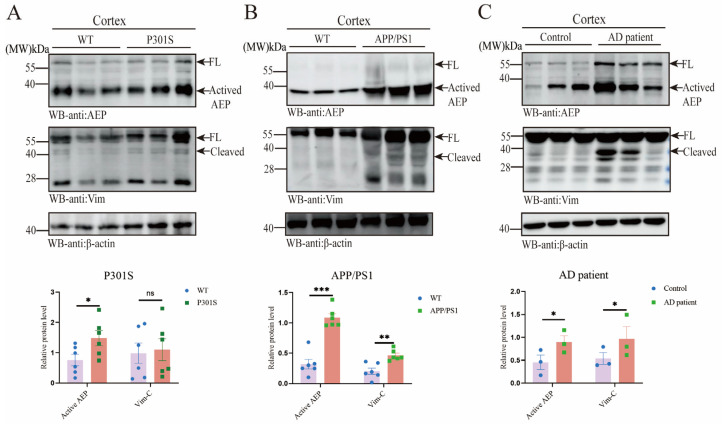

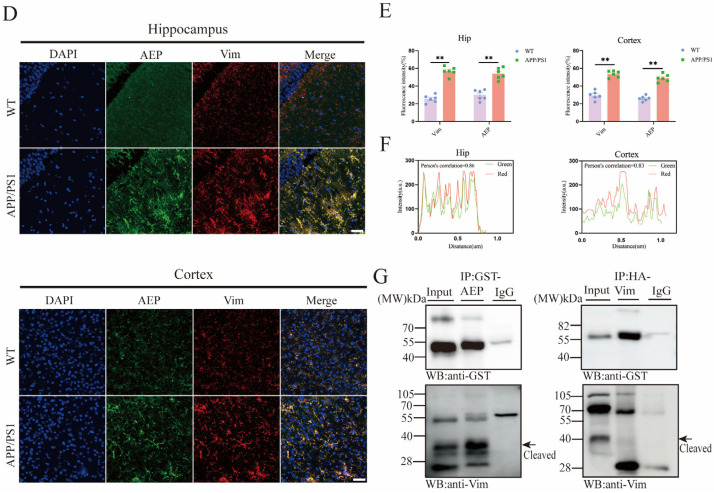
AEP and Vim escalate in AD brain. (**A**–**C**) Western blot analysis revealed the protein levels of AEP and Vim in cortex brain tissues from APP/PS1 mice, P301S mice, and AD patients. Quantitative analysis of AEP and Vim protein level. Data are mean ± SEM; *n* ≥ 3; * *p* < 0.05, ** *p* < 0.01, *** *p* < 0.001 by one-way ANOVA. (**D**,**E**) Immunohistochemistry demonstrates the expression patterns of AEP and Vim in brain sections from APP/PS1 and WT mice. Scale bar: 20 μm. Quantitative analysis. Data are mean ± SEM; *n* ≥ 3; ** *p* < 0.01 by one-way ANOVA. (**F**) ImageJ analysis revealed a colocalization correlation coefficient of 0.86 for AEP and Vim in the hippocampus and 0.83 in the cortex. (**G**) The co-immunoprecipitation (Co-IP) was employed to detect the interaction between exogenous GST-AEP and HA-Vim-EGFP.

To elucidate the relationship between AEP and Vim in Alzheimer’s disease (AD), we conducted co-validation studies in AD mouse models. Our results demonstrated that AEP, in its activated state, was significantly up-regulated in both APP/PS1 and P301S mice (Figure 1A,B). However, Vim expression was only notably elevated in APP/PS1 mice. Additionally, Vim fragmentation bands were detected in the cortex of APP/PS1 mice, but were absent in P301S mice (Figure 1A,B). To further corroborate these findings, we analyzed brain tissues from AD patients and observed results consistent with those from APP/PS1 mice (Figure 1C). Moreover, utilizing the AlzData and GEO databases, we examined Vim expression across various brain regions in AD and normal cohorts. The analysis revealed significant upregulation of Vim in the entorhinal cortex, hippocampus, and temporal cortex, while no significant differences were observed in the frontal cortex (Supplementary Figure S1A). Next, we explored the morphological relationship between AEP and Vim. We stained brain sections from the cortex and hippocampus of APP/PS1 and P301S mice (Figure 1D, Supplementary Figure S1C,D) to observe the spatial relationship between AEP and Vim. Immunofluorescence analysis in APP/PS1 mice showed significant upregulation of both AEP and Vim (Figure 1E). Fluorescence colocalization analysis using Fiji ImageJ-win64(2024) revealed a Pearson correlation coefficient greater than 0.5, indicating a substantial interaction between AEP and Vim (Figure 1F). To further validate this interaction, we performed co-immunoprecipitation (Co-IP) assays (Figure 1G). The Co-IP results confirmed that AEP interacts with Vim (Figure 1G). Molecular docking studies provided insights into the potential interaction sites between AEP and Vim and generated an interaction network diagram based on database analysis (Supplementary Figure S1B). Specifically, ASP-367, ASN-371, and GLU-374 in the Vim structure were identified as interaction sites for Vim, while GYS-378, GLY-375, GLU-413, LYS-347, and LYS-351 in the AEP structure were identified as interaction sites for AEP. Additionally, AEP-TYP-433 interacts with Vim-ASP-451. In summary, our findings indicate that AEP and Vim are upregulated in AD, mediating Vim fragmentation. The interaction between AEP and Vim was confirmed through Co-IP and molecular docking studies.

### 2.2. Vim Is a Substrate of AEP, Cleaved at N283 Residue

**Figure 2:**
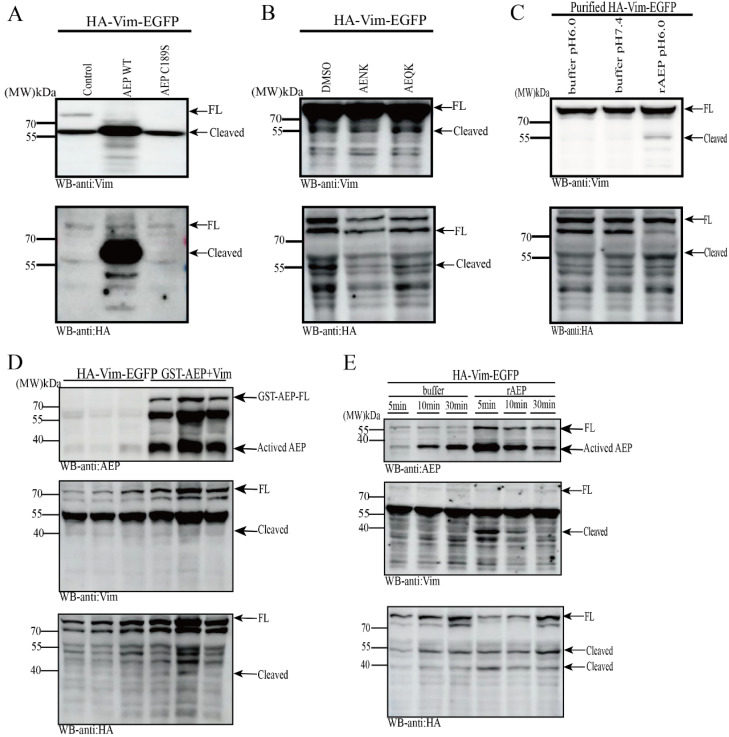

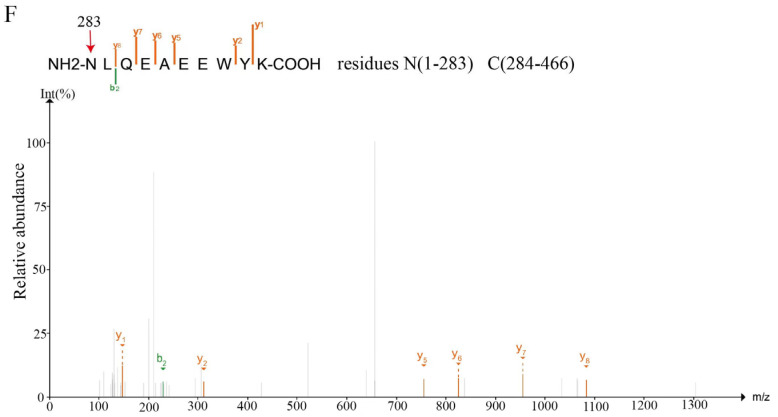
Vim is a substrate of AEP, cleaved at N283 residue. (**A**) Vim cleavage assay with enzymatic-dead AEP. HA-Vim-EGFP was co-transfected with wild-type or C189S mutant myc-AEP in HEK293 cells, Western blot analysis of cleavage of Vim. (**B**) Vim cleavage assay with AEP inhibitor AENK peptide. HA-Vim-EGFP was transfected into HEK293 cells 48 h later, DMSO or AENK or AEQK 5 nM peptide (AEQK peptide was employed as a control) was added, incubated for 30 min, and a Western blot analysis was conducted on the cleavage of Vim. (**C**) Vim cleavage assay with recombinant AEP (rAEP). Buffer with different pH values were used for Vim cleavage assay in vitro, Western blot analysis of cleavage of Vim. (**D**) After co-transfection of HEK293 cells with Vim or Vim and AEP for 48 h, the supernatant of cell lysis were collected for WB assay, and significant Vim fragments were found in the Vim and AEP systems. (**E**) After transfecting HEK293 cells with Vim plasmid for 48 h, cell supernatants were collected and incubated with recombinant AEP (rAEP) for different time periods in vitro, and Western blot analysis of AEP-cleaved Vim. (**F**) Vim was cleaved at N283 residue. HA-Vim-EGFP was transfected into HEK293 cells for 48 h, cell lysis was incubated with pH 6.0 buffer for 30 min. Then, the Vim fragment band was obtained by Coomassie brilliant blue staining after collecting the cell lysis, Interpretation of the b-ion series and y-ion series provided the peptide sequence N-LQEAEEWYK. The Vim fragment site was confirmed by mass spectrometry analysis.

AEP is significantly activated on AD and cleaves a variety of substrates, promoting their deposition and aggravating AD or PD pathology [20,21,22,23]. Previously, we discovered that Vim exhibits fragmented bands in the AEP-activated state in the brains of both APP/PS1 mice and Alzheimer’s disease (AD) patients. Additionally, the interaction between AEP and Vim has been thoroughly elucidated both in vitro and in vivo. Moving forward, we aim to determine whether the Vim fragment bands are a result of AEP-mediated cleavage. To this end, proteins derived from HEK-293 cells were collected in vitro and incubated with recombinant AEP (rAEP was activated using our experimental method). Two groups of Vim were then detected using HA, EGFP, and Vim antibodies to assess whether they were cleaved by rAEP. The results indicated that Vim fragmentation was more pronounced in the co-transfection system of AEP and Vim. However, a small number of Vim fragments were also observed in the Vim transfection system alone (Figure 2D). We speculate that this may be due to endogenous AEP in HEK-293 cells cleaving Vim. Previous research has demonstrated that AEP exhibits time-dependent cleavage of its substrates [24]. To further validate the time-dependent cleavage, we conducted experiments. Vim proteins were collected in vitro and co-incubated with either activated rAEP or AEP buffer solution. SDS denaturing solution was added to the system for subsequent Western blot (WB) analysis. The results revealed that sequential and increasing cleavage bands appeared in the system incubated with Vim and rAEP at different time intervals (Figure 2E). Additionally, we performed experiments using wild-type AEP and mutant AEP with a functional site alteration at position 189 to reconfirm the cleavage of Vim by AEP. The Vim protein collection method remained consistent with the previous approach, and detection was carried out using Vim, HA, and EGFP antibodies, separately. WB analysis showed that Vim cleavage was most prominent in the AEP wild-type (WT) group (Figure 2A). Although some fragment bands were observed in the AEP C189S mutant system, the potential role of endogenous AEP cannot be overlooked. A significant inhibitory effect of the AEP inhibitor AENK (AEP inhibitory peptide, Ala-Glu-Asn-Lys) on AEP activity was observed in experiments using AENK (AEQK, AEP control peptide, Ala-Glu-Gln-Lys) (Figure 2B). To further elucidate the cleavage effect of AEP on Vim, we purified HA-Vim-EGFP protein and co-incubated it with rAEP in vitro. Significant cleavage was observed in the rAEP and Vim co-incubation groups (Figure 2C). After confirming the cleavage of Vim by AEP using various methods, we performed mass spectrometry analysis on Coomassie brilliant blue-stained gels to pinpoint the cleavage site of AEP on Vim (Figure 2F). The results indicated that the size of the Vim fragment detected by WB corresponded to Vim-N283. These data suggest that Vim is cleaved by AEP at the N283 site, producing Vim-N(1–283) and Vim-C(284–466) fragments.

### 2.3. Vim Mainly Localized in APP/PS1 Mice Hippocampus Astrocytes, and Its Increase Precedes the Development of Aβ Pathology

**Figure 3:**
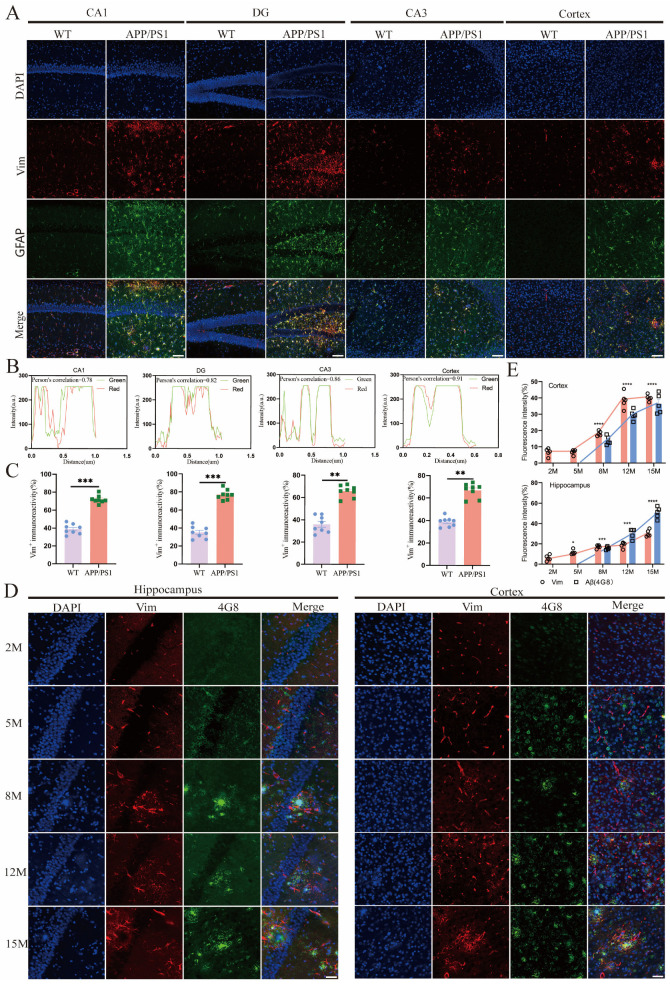
Vim is mainly localized in APP/PS1 mice hippocampus astrocytes, and its increase precedes the development of Aβ pathology. (**A**) Immunohistochemistry demonstrated that Vim mainly colocalized with GFAP in various brain regions, including the CA1, CA3, DG, and the cortex, in both APP/PS1 and WT mice. Scale bar: 40 μm. (**B**) ImageJ analysis revealed a colocalization correlation coefficient of 0.78 for Vim and GFAP in the CA1, 0.82 in the DG, 0.86 in the CA3, and 0.91 in the cortex. (**C**) Quantitative analysis of Vim expression in 12-month-old WT and APP/PS1 mice. Data are mean ± SEM; *n* ≥ 3; ** *p* < 0.01, *** *p* < 0.001 by one-way ANOVA. (**D**,**E**) Vim and Aβ pathology increased in APP/PS1 mice in an age-dependent manner. Immunofluorescence analysis of Vim and Aβ pathology in cortex and hippocampus in APP/PS1 at gradient age series. Scale bar: 40 μm. Quantitative analysis of Vim and Aβ expression in WT and APP/PS1 mice. Data are mean ± SEM; *n* ≥ 3; * *p* < 0.05, *** *p* < 0.001, **** *p* < 0.0001 by one-way ANOVA.

Further studies in chickens and mice have demonstrated that Vim mRNA is predominantly expressed in connective tissue and central nervous system cells, as well as in red blood cells and muscle cells [25,26]. Additionally, Vim has the capacity to translocate from the pericellular region to the nucleus, where it forms an intricate network structure [27]. Next, we will investigate the cellular localization of Vim in the central nervous system. To address this question, we plan to conduct studies in two AD mouse models: APP/PS1 and P301S. Accordingly, we performed immunofluorescence double staining for the markers of neurons (NeuN), astrocytes (GFAP), microglia (Iba-1), and Vim in these cell types (Figure 3A; Supplementary Figure S2A,B). The results indicated that in APP/PS1 mice, across the CA1, CA3, DG regions, and cortex, Vim expression was relatively low in neurons and Iba-1. However, it exhibited a high degree of colocalization with GFAP (with a Pearson coefficient significantly greater than 0.5) (Figure 3B). Additionally, we quantitatively analyzed the fluorescence intensity of Vim in WT and APP/PS1 mice, revealing that Vim expression was significantly higher in APP/PS1 mice compared to WT mice (Figure 3C). Consistent results were also observed in another AD model, P301S mice (Supplementary Figure S3A–C). These findings align with previous studies, suggesting that Vimentin may be associated with the organization of the GFAP network, thereby rendering it responsive to disease or inflammation [28]. Although Vim has been reported to be re-expressed and redistributed by neurons in AD [29], we did not observe this phenomenon in our study. Next, we aim to investigate the relationship between the upregulation of Vim expression and AD pathology. In APP/PS1 mice at different ages, Vim was detected at 2 months and gradually increased over time. In contrast, Aβ pathology appeared at 8 months, suggesting that Vim may serve as an upstream regulator of Aβ pathology (Figure 3D,E). Additionally, we examined the pathological Tau protein in AD models. Vim has been reported as a cytoskeletal component involved in microtubule-associated proteins, with a direct interaction between Vim and Tau [30]. However, this interaction has not been validated in vivo, and we did not observe any interaction between Vim and Tau in our study. Immunofluorescence results showed no colocalization between Vim and either Tau5 or AT8, indicating that Vim and Tau pathology are not closely related in terms of spatial distribution (Supplementary Figure S3E,F). In summary, our data demonstrate that Vim is highly expressed in astrocytes and functions as an upstream factor of Aβ pathology.

### 2.4. Vim-Positive Astrocytes in APP/PS1 Mice Were More Likely to Cluster Around Aβ Plaques

**Figure 4:**
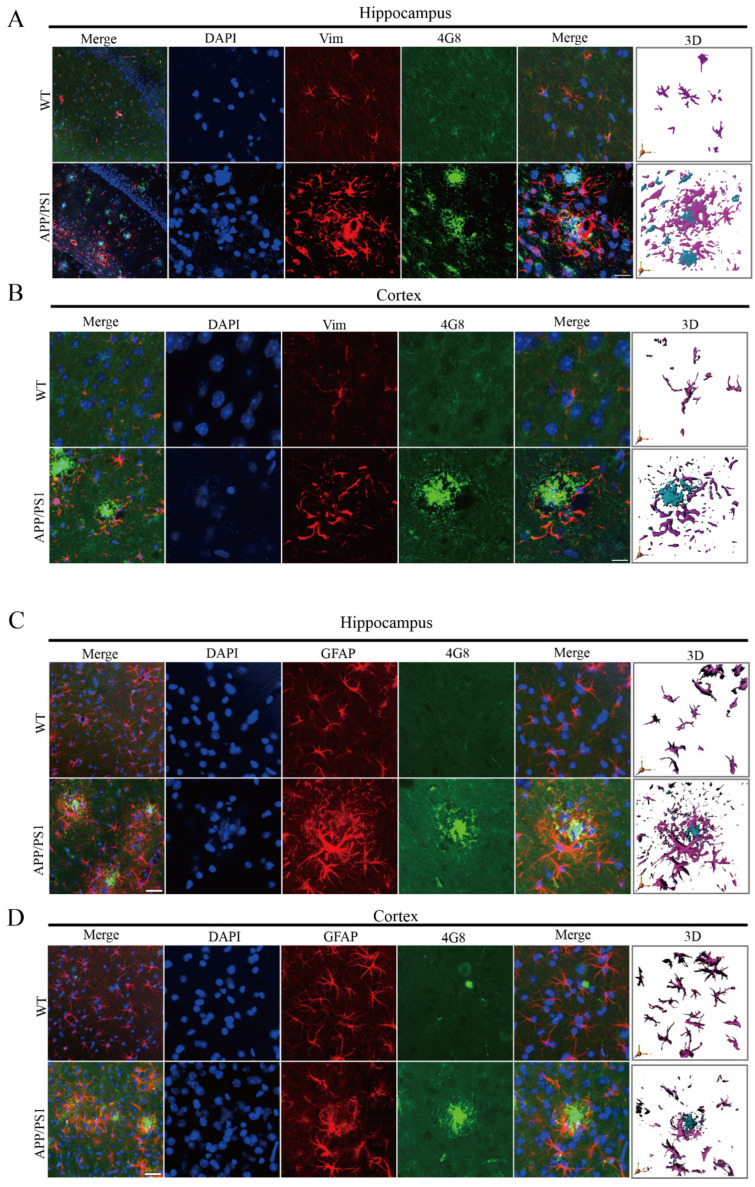
Vim-positive astrocytes in APP/PS1 mice were more likely to cluster around Aβ plaques. (**A**,**B**) Immunohistochemistry revealed that the pathological association between Vim and Aβ was localized in brain sections (hippocampus and cortex) from 12-month-old APP/PS1 and wild-type (WT) mice. Additionally, three-dimensional (3D) reconstruction was employed to analyze the distribution of Vim around Aβ plaques. Scale bar: 10 μm. (**C**,**D**) Immunohistochemistry revealed that the pathological association between GFAP and Aβ was localized in brain sections (hippocampus and cortex) from 12-month-old APP/PS1 and WT mice. Additionally, 3D reconstruction was employed to analyze the distribution of Vim around Aβ plaques. Scale bar: 10 μm.

The results described above demonstrate that Vim exhibits significant colocalization with astrocytes, which are the most abundant glial cells in the nervous system. Astrocytes respond to neurodegenerative disorders through astrogliosis, characterized by converting to a reactive inflammatory state and becoming active in response to Aβ during AD [31,32]. To explore how Vim affects Aβ pathology, we used the 4G8 antibody and Vim for immunofluorescent co-staining of the hippocampus and cortex in APP/PS1 mice (Figure 4A,B). The results showed increased Vim and Aβ plaque accumulation in both the cortex and hippocampus. We also observed that Vim surrounds Aβ plaques (Figure 4A,B). To better illustrate this phenomenon, we employed 3D reconstruction to clearly depict the relationship between Vim and Aβ (Figure 4A,B, right). Similar aggregation patterns were also observed between GFAP and Aβ (Figure 4C,D).

Reactive astrocytes are characterized by increased GFAP expression and the polymerization of many intermediate filaments (IFs) [33]. GFAP and Vim form an important network structure. Co-increased expression of GFAP and Vim has only been reported in cells near injury sites [34]. In injured regions, three-fifths of astrocytes co-express Vim and GFAP, while the remainder are only GFAP-positive [35]. When astrocytes are cultured with Vim, the IF network undergoes reorganization [36]. In vivo experiments have shown that during development, the expression of Vim and GFAP in the astrocyte lineage is strictly regulated: IFs containing Vim are first expressed in astrocyte precursors, followed by co-expression of Vim and GFAP, and finally, in adults, two differentiated astrocyte populations emerge—one expressing only GFAP, and the other co-expressing GFAP and Vim. Similarly, in pathological conditions, some astrocytes upregulate GFAP and Vim following CNS injury. To address potential issues with the network structure formed between Vim and GFAP, researchers have identified significant flaws in this network structure in Vim^−/−^ models [37]. In our study, we aimed to investigate whether AEP-mediated fragmentation of Vim affects the network structure between Vim and GFAP and how fragmented Vim mediates Aβ phagocytosis by astrocytes. To address these questions, we transfected human-passaged astrocyte cells (SW1783) with Vim fragments (Supplementary Figure S4A). Immunofluorescence results showed that both the Vector group and Vim-FL-transfected SW1783 cells exhibited phagocytosis of exogenous Aβ. However, this phenomenon was not observed in Vim-N- and Vim-C-transfected cells. Interestingly, fragmented Vim-transfected SW1783 cells not only lost phagocytic ability, but also exhibited morphological disorganization (Supplementary Figure S4A). Specifically, no distinct GFAP cell outlines were observed in Vim-N- or Vim-C-transfected cells. These results suggest that Vim fragmentation affects astrocyte phagocytosis and the formation of the network between GFAP and Vim. Based on these observations, we plan to construct molecular models of Vim-FL, Vim-N, and Vim-C and use molecular docking technology to determine whether Vim and GFAP interact (Supplementary Figure S4B,C). The results indicate successful docking only between Vim-C and GFAP (Supplementary Figure S4C). The detailed interaction sites between Vim-C and GFAP are demonstrated, suggesting that the interaction between Vim-C and GFAP may disrupt the network structure between Vim and GFAP, leading to impaired phagocytic function in astrocytes. It is important to note that while immunofluorescence staining of Vim-N-transfected SW1783 cells did not reveal GFAP morphology, molecular docking did not predict an interaction between Vim-N and GFAP. In conclusion, our data suggest that Vim responds to pathological Aβ plaques in APP/PS1 mice by wrapping around Aβ plaques. Moreover, fragmented Vim (especially the Vim-C terminal) affects the phagocytosis of extracellular Aβ and leads to morphological changes in astrocytes.

### 2.5. Vim Fragmentations Promote Cell Apoptosis and Aβ Aggregation via ApoE Downregulation

**Figure 5:**
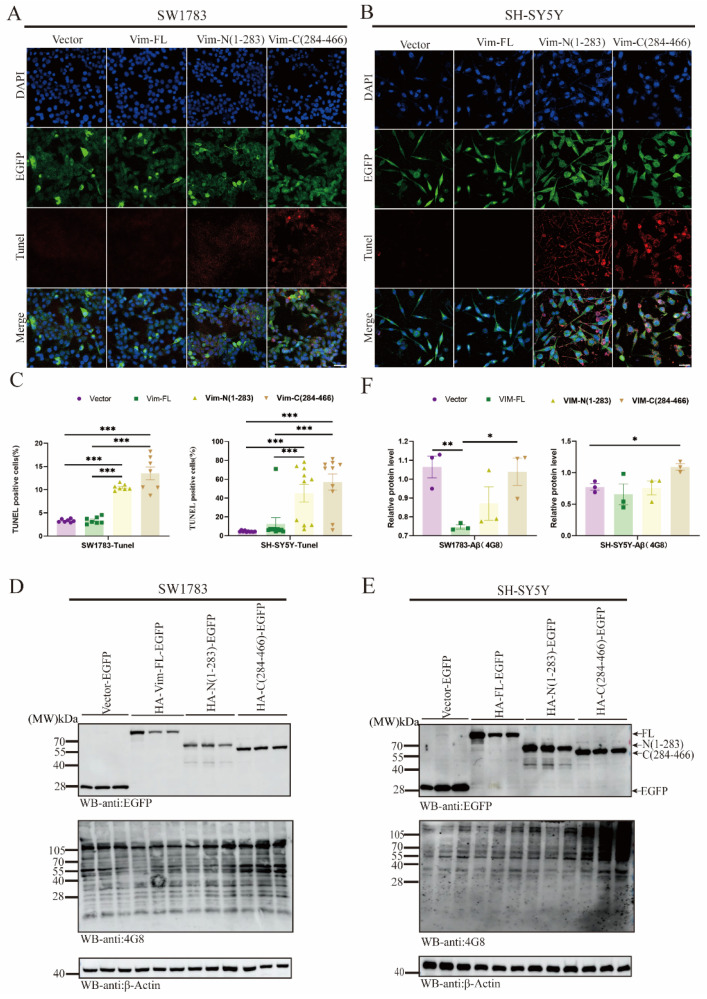
Vim fragmentation promotes cell apoptosis and Aβ aggregation. (**A**,**B**) TUNEL assay on SW1783 and SH-SY5Y cells. Colocalization of TUNEL and EGFP in cells. Confocal immunofluores-cence microscopy was performed to detected vector-, Vim-FL-, Vim-N(1–283)-, or Vim-C(284–466)-transfected SW1783 and SH-SY5Y cells. Scale bar, 10 μm. (**C**) Quantification of TUNEL-positive cells. Data are mean ± SEM; *n* ≥ 3; *** *p* < 0.001 by one-way ANOVA. (**D**,**E**) Western blot analysis was per-formed to examine the levels of Aβ in the presence of different Vim fragments in SW1783 and SH-SY5Y cells. (**F**) Quantitative statistical analysis was conducted to determine the protein levels of Aβ. Data are mean ± SEM; *n* ≥ 3; * *p* < 0.05, ** *p* < 0.01 by one-way ANOVA.

Next, we constructed a Vim fragmentation plasmid to explore the specific functions of the N-terminus and C-terminus. Initially, SH-SY5Y cells were transfected with fragmented Vim plasmids, Vim-FL, and controls. MTT assays were used to assess neurotoxicity (Supplementary Figure S5A), revealing that Vim-N/C significantly increased cell mortality in SH-SY5Y cells compared to the Vim-FL group. Additionally, TUNEL assays, consistent with MTT results, were performed in both SW1783 and SH-SY5Y cells (Figure 5A–C). Given previous findings that Vim is significantly associated with Aβ pathology, we aimed to investigate how Vim fragmentation (Vim-N/C) regulates Aβ at the protein level. SH-SY5Y and SW1783 cells were transfected with Vim fragment plasmids, and Vim-C was found to be significantly upregulated compared to the control group (Figure 5D,E). However, the mechanistic pathway underlying fragmented Vim-mediated upregulation of Aβ remains unclear. Monoamine oxidase (MAO) exists in two forms, MAO-A and MAO-B, each with distinct functions. MAO-B deaminates exogenous amines and certain dopamine molecules. MAO-B levels increase with age in various regions of the human brain, including the neocortex, hippocampus, caudate nucleus, hypothalamus, substantia nigra, thalamus, and amygdala [38]. MAO-B is highly abundant in astrocytes, and its activity is upregulated during neuroinflammatory processes [39]. Additionally, MAO-B in reactive astrocytes can secrete large amounts of the inhibitory neurotransmitter γ-aminobutyric acid (GABA), which is transported to the dentate gyrus (DG) region of the hippocampus, leading to synaptic plasticity impairment and cognitive deficits [40]. Activated MAO is also involved in the sequential cleavage of amyloid precursor protein (APP) by β-secretase and γ-secretase, contributing to Aβ aggregation [41]. Another critical molecule, apolipoprotein E (APOE), has three isoforms: E2, E3, and E4. APOE is predominantly produced by astrocytes and delivers cholesterol and other essential lipids to neurons via low-density lipoprotein receptor (LDLR) family members [42]. Recent studies have highlighted the protective role of E3 in AD [43]. Overall, astrocyte-derived ApoE is crucial for Aβ transport and aggregation. To elucidate whether fragmented Vim-upregulated Aβ is associated with MAO-B or ApoE, we measured MAO-B and ApoE protein levels at the cellular level (Supplementary Figure S5B,C). Results showed no significant difference in MAO-B levels between Vim-FL and Vim-N/C groups. However, ApoE was significantly downregulated in Vim-C. These findings underscore the toxic effects of the Vim-C terminus, including impaired phagocytosis, disruption of the Vim-GFAP network, increased apoptosis, and upregulation of Aβ levels. These results suggest that Vim-C may serve as a potential therapeutic target in AD.

### 2.6. Vim-N283A Mutation Reverses Cell Apoptosis and Prevents Aβ Aggregation by Restoring ApoE Levels

**Figure 6:**
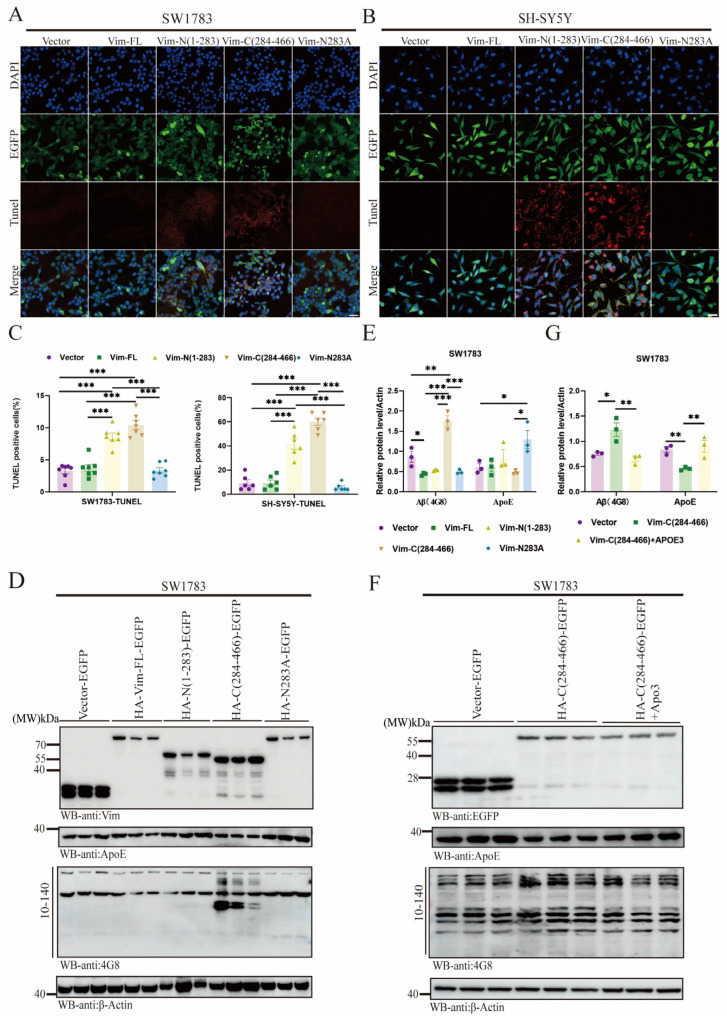
Vim-N283A mutation reverses cell apoptosis and prevents Aβ aggregation by restoring ApoE levels. (**A**,**B**) TUNEL assay on SW1783 and SH-SY5Y cells. Colocalization of TUNEL and EGFP in cells. Confocal immunofluorescence microscopy was performed to detected vector-, Vim-FL-, Vim-N(1–283)-, Vim-C(284–466)-, or Vim-N283A-transfected SW1783 and SH-SY5Y cells. Scale bar: 10 μm. (**C**) Quantification of TUNEL-positive cells. Data are mean ± SEM; *n* ≥ 3; *** *p* < 0.001 by one-way ANOVA. (**D**,**E**) Western blot analysis was performed to examine the levels of Aβ and ApoE in the presence of different Vim fragments or Vim-N283A in SW1783 cells. Quantitative statistical analysis was conducted to determine the protein levels of Aβ and ApoE. Data are mean ± SEM; *n* ≥ 3; * *p* < 0.05, ** *p* < 0.01, *** *p* < 0.001 by one-way ANOVA. (**F**,**G**) Western blot analysis was performed to examine the levels of Aβ and ApoE in the presence of Vim-C fragment with/without exogenous ApoE3 recombinant protein in SW1783 cells. Quantification of ApoE and Aβ level, Data are mean ± SEM; *n* ≥ 3; * *p* < 0.05, ** *p* < 0.01 by one-way ANOVA.

We have previously observed cytotoxic effects mediated by Vim fragmentation, particularly the Vim-C terminal. Next, we plan to mutate asparagine (N) at Vim-283 to alanine (A) using site-directed mutagenesis (Supplementary Figure S6). To assess the impact of Vim on cell function, we transfected the Vim-N283A plasmid into SH-SY5Y and SW1783 cells and detected apoptosis using the TUNEL assay. The results showed that apoptosis was significantly reduced after the Vim-N283A mutation (Figure 6A–C). This reversal was attributed to the site-directed mutation at Vim-283. Protein level analysis clearly demonstrated that Vim-N283A mutation increased ApoE release, thereby reducing Aβ levels (Figure 6E). The Vim-C fragment exhibited a significant detrimental effect on ApoE levels. To counteract this toxic effect, we supplemented the Vim-C group with exogenous ApoE3 (rApoE3). Western blot (WB) results indicated that recombinant ApoE3 mitigated the toxic effects (Figure 6F,G). These findings suggest that the Vim-C fragment impairs ApoE release from astrocytes, leading to Aβ accumulation.

## 3. Discussion

Vim is an intermediate filament protein that plays a crucial role in the central nervous system. Research has shown that Vim has both beneficial and detrimental effects. On the positive side, Vim has been associated with a reduction in pathological neovascularization, decreased photoreceptor cell death and monocyte infiltration in retinal degeneration, improved post-traumatic axonal and synaptic regeneration, better integration of neural grafts, increased differentiation of transplanted cells, enhanced sensorimotor neuronal connections, altered global functional connectivity, and improved functional recovery after ischemic stroke, despite increased glial and axonal plasticity responses [44,45,46]. However, the dysregulation of Vim has been implicated in the development and progression of diseases, including major neurodegenerative disorders such as Alzheimer’s disease (AD) and Parkinson’s disease (PD). Both full-length and cleaved forms of citrullinated Vim were detected in the cerebral cortices of patients with sporadic Creutzfeldt–Jakob disease (sCJD). The presence of citrullinated Vim was primarily confirmed in reactive astrocytes that were positive for Vim, GFAP, and YKL-40. Biochemically, citrullination enhanced the resistance of Vim to fragmentation mediated by caspase-3 and caspase-9 [11].

This study identified Vim as a novel substrate of AEP, which cleaves Vim at the N283 residue, generating N-terminal (1–283) and C-terminal (284–466) fragments. The findings demonstrated that AEP-mediated Vim proteolysis increases cell mortality and impairs the phagocytic function of astrocytes toward Aβ plaques by decreasing ApoE levels, leading to increased extracellular Aβ aggregation and synaptic toxicity. Additionally, the study found that Vim-positive astrocytes in APP/PS1 mice cluster around Aβ plaques, and that the Vim-N283A mutation reverses cell apoptosis and prevents Aβ aggregation by restoring ApoE levels.

Reactive astrocytes and Aβ-containing astrocytes are common manifestations in the brains of Alzheimer’s disease (AD) patients [47]. Astrocytes are believed to play a role in clearing Aβ from the brain parenchyma into the perivascular space, across the blood–brain barrier or through enzymatic degradation. A mixture of amyloid-associated proteins (AAPs) has been found to modify Aβ uptake by human astrocytes [48]. Although few studies have reported changes in Aβ plaque burden following simultaneous knockout of GFAP and Vim, the specific mechanisms by which GFAP and Vim affect Aβ plaques remain unclear.

It is well established that a protein’s structure determines its functional role. Research has analyzed the structure of Vim, which consists of 310 amino acids in a central region, with a coil 1A motif and an α-helical linker L1 at the N-terminus. The C-terminus includes the coil 1B motif and the coil 2 motif, connected by a short β linker L12. The coil 2 motif is further divided into coil 2A and coil 2B motifs, linked by an L2 structure [49,50,51,52,53]. Interestingly, the coil 2B motif structure aligns with the Vim-C (284–366) fragment, resulting from AEP cleavage in this study (Supplementary Figure S7). The N-terminal and C-terminal regions of coil 2 contain conserved repeats between Vim proteins, and this segment sequence may be crucial for the formation of Vim in response to mechanical stress [54,55]. Understanding the structure of Vim allows us to better explore the functional role of Vim-C, but the mechanism by which Vim-C alters GFAP morphology in this study remains unclear and will be further investigated in our follow-up studies.

Additionally, we observed in vivo Vim aggregation surrounding Aβ plaques. Constructs of Vim-N/C-fragmented plasmids transfected into cells showed increased apoptosis rates and significant upregulation of Aβ levels. After mutating Vim-283 from N to A, apoptosis was reversed, and Aβ levels decreased. Simultaneously, ApoE expression was detected, revealing upregulation of Vim-N283A. Therefore, we conclude that AEP-mediated Vim fragmentation produces Vim-N/C fragment proteins with cytotoxic effects, particularly the Vim-C terminal, which not only disrupts GFAP morphology in astrocytes, but also impairs their phagocytic function. It also reduces ApoE protein release, impairing Aβ clearance and transport, ultimately leading to increased Aβ levels.

It is worth noting that the three subtypes of ApoE: E2, E3, and E4. E2 is protective, and E4 serves as one of the most risk factors for sporadic AD (sAD) [56]. E3’s role lies between these two [57]. ApoE forms copolymers with Aβ containing lipidated ApoE, accelerating its clearance and avoiding Aβ-induced toxicity [58]. This suggests that increased ApoE lipidation facilitates Aβ clearance or transports it to intracellular lysosomes or ubiquitin proteasomes for degradation [59]. Previously, the role of E3 isoforms was relatively obscure, but recent studies have confirmed the protective role of ApoE3 [43]. In our study, Vim-C-mediated reduction in ApoE release from astrocytes led to increased Aβ levels, and supplementation with exogenous ApoE3 rescued this phenotype. However, the mechanism by which Vim-C affects ApoE release remains unclear. Current research on ApoE in AD focuses on its receptors, lipidation, and structure [60]. Thus, our future research will also explore these areas. Moreover, we did not validate the Vim fragment in vivo through the ApoE regulation of the Aβ pathway. In future research, we will focus on addressing these shortcomings and further explore the unique interaction between Vim-C and GFAP and its impact on astrocyte phagocytosis. In summary, this study newly identifies Vim as a substrate for AEP and elucidates the relationship and potential mechanisms of fragmented Vim in Aβ pathology, providing a new target or direction for AD intervention.

Proteomic analyses in PD patients revealed calpain-mediated cleavage of vimentin at the N-terminal domain, producing variably truncated N-terminal isoforms. Remarkably, levels of these N-truncated vimentin forms were significantly reduced in PD fibroblasts, potentially attributable to oxidative stress and elevated intracellular calcium levels pathological features observed in fibroblasts from two PD patients [61]. These findings indicate that extensive proteolytic degradation of vimentin compromises its physiological functions and may play a pivotal role in PD pathogenesis [62]. It should be emphasized that the mechanistic role of the Vimentin-C fragment identified in this study remains unclear in PD, and future investigations will elucidate this pathway. In other neurodegenerative disorders, such as amyotrophic lateral sclerosis (ALS), co-localization of TDP-43 (a hallmark pathological protein in ALS) and vimentin was observed in dermal fibroblasts through immunophenotyping [63]. Post-translational modification of vimentin via citrullination has been reported to critically contribute to multiple sclerosis (MS) and AD pathology. In MS, reactive astrocytes exhibit upregulated peptidylarginine deiminase 4 (PAD4, a key citrullination catalyzing enzyme), leading to aberrant citrullination of myelin basic protein (MBP) and vimentin [64]. Recent studies further identified extensively citrullinated Aβ plaques in AD brains, highlighting citrullinated molecules as promising therapeutic targets for AD mechanism exploration [65].

Collectively, both full-length and truncated vimentin isoforms exert significant pathophysiological impacts across neurodegenerative disorders. Elucidating these mechanistic pathways will facilitate the development of targeted strategies to modulate disease progression and pathogenesis.

## 4. Materials and Methods

### 4.1. Mice

APP/PS1, P301S mice were provided by this experimental multiplication, and the genotype mice were determined by rat tail gene identification. In accordance with the requirements of the Institutional Animal Care and Use Committee, we reduced the number of animals to a minimum when the experimental conditions were the same. All mice were maintained under specific pathogen-free (SPF) conditions on a 14 h light/10 h dark cycle with free access to food and water. The experimental procedures were approved by the laboratory animal management of Jianghan University. Mice were randomly divided into different groups using a random number table. During animal experiments, the investigator was blinded to group allocation.

### 4.2. Western Blot Analysis

Mouse brain tissue samples, cells, or human brain tissue were lysed in lysis buffer (50 mM Tris, pH 7.4, 40 mM NaCl, 1 mM EDTA, 0.5% Triton X-100, 1.5 mM Na_3_VO_4_, 50 mM NaF, 10 mM sodium pyrophosphate, and 10 mM sodium β-glycerophosphate, supplemented with protease inhibitor cocktail) and centrifuged at 13,000× *g* for 10 min. The supernatant was boiled in an SDS loading buffer. After SDS-PAGE, the sample was transferred to a nitrocellulose membrane (Catalog No: AR0135-04, 0.45 μm). The membrane was incubated with the primary antibody overnight at 4 °C. Membranes were washed 3 times in TBST and incubated with HRP-conjugated secondary antibodies (Catalog No: BA1050). The signal was generated using an enhanced fluorescence (ECL) substrate (Catalog No: E370604). Experiments were repeated at least three times and treated in parallel.

### 4.3. Immunofluorescence

Animals were cardiac perfused with 4% paraformaldehyde (PFA) in 0.1 M phosphate-buffered saline (PBS) (pH 7.4) under anesthesia with isoflurane and chloral hydrate (3%). Harvest brains were post-fixed overnight at 4 °C in the same fixative, dehydrated with 30% sucrose in PBS, and sliced serially on a 30 μM Mona slide microtome (Leica, Wetzlar, Germany). For immunofluorescence, sections are permeabilized in PBS/0.1% Triton X-100 for 10 min and blocked with PBS/0.1% Triton X-100 along with normal bovine serum for 1 h at room temperature. The sections were then incubated with primary antibody in 2% serum in PBS/0.1% Triton X-100 overnight at 4 °C. Sections were then washed and incubated for 1 h at room temperature with Alexa Fluor 488-, Alexa Fluor 594-, or Alexa Fluor 647-conjugated secondary antibody (Invitrogen, Carlsbad, CA, USA). After washing with PBS, sections were incubated with DAPI (Catalog No: P0131-25 mL) to stain nuclei. Images were captured using a laser scanning confocal microscope (Purchased from Leica Microsystems (Shanghai) Trading Co., Ltd., Shanghai, China) (Leica, the confocal microscopy, was performed using 20× and 40× immersion lens).

### 4.4. Cell Culture and Transduction

SH-SY5Y cells, human-passaged astrogel cells (SW1783), as well as N2a cells, were cultured in Dulbecco’s modified Eagle medium (DMEM) containing 10% fetal bovine serum and penicillin/streptomycin at 37 °C and 5% CO_2_. For transfection, the transfection system was prepared by mixing 100 μL of OptiMEM4 μL, lipofectamine-2000 (Invitrogen), 4 μL of DNA in a 1.5 mL EP tube and the mixture was added to the medium. Aβ-594 treatment was given 24 h later, and cell proteins were collected after 48 h of incubation at 37 °C and 5% CO_2_.

### 4.5. Human Tissue Sample

Brain samples were obtained from the Chinese Brain Bank Center at the Zhong Nan University of Science and Technology, including 6 samples from 3 patients who died of AD (1 males and 2 females, 85 ± 10 years old) and 3 controls (1 males and 2 females, 85 ± 10 years old). Brain samples were obtained with written consent from the donor’s family members. This experiment was approved by the Medical Ethical Committee of Jiang Han University (approval JHDXLL2022-043).

### 4.6. In Vitro Vim Cleavage Assay

The Vim cleavage assay was assessed as described previously. HEK-293 cells were transfected with Vim plasmids. Then, 48 h after transfection, the cells were collected, washed, lysed in AEP lysis buffer (50 mM sodium citrate, 5 mM DTT, 0.1%CHAPS, and 0.5% Triton X-100, pH 5.5), and then centrifuged for 15 min at 14,000× *g* at 4 °C. The supernatants were incubated with recombinant AEP (5 μg/mL) at pH 6.0 and 37 °C. The samples were then boiled in 1× SDS loading buffer and analyzed by immunoblotting.

### 4.7. Co-IP Assay

HA-Vim-EGFP and GST-AEP plasmids were transfected into HEK-293 cells for 48 h. Cells were washed twice with PBS and lysed on ice for 20 min using RIPA lysis buffer (high strength) supplemented with 1× phosphatase inhibitors, 1× protease inhibitors, and 1× PMSF. The lysates were collected and subjected to sonication (100 W power, 5 s pulses with intervals) to disrupt cellular structures. The lysates were centrifuged at 13,000 rpm for 20 min at 4 °C to pellet debris. The resulting protein supernatants were incubated overnight at 4 °C with either anti-vimentin (Vim) or anti-GST antibodies to form antibody–protein complexes. The following day, magnetic beads (e.g., protein A/G beads) were washed three times with PBS (5 min per wash). The antibody–protein complexes were then incubated with the pre-washed beads at room temperature for 4 h. After incubation, the beads were washed three times with PBST (PBS containing 0.1% Tween-20, 5 min per wash) to remove nonspecific bindings, and then boiled in 2× SDS loading buffer for 10 min to elute bound proteins. Samples were analyzed by Western blotting.

### 4.8. Vim Fragmentation Plasmid Construction

Vim fragments and mutated plasmids were constructed using homologous recombination, and vector primers (F1: 5′-GCGGATCCACCGGTCATG-3′ R1: 5′-GTAATCTGGTACGTCGTATGGGGTACA-3′), Vim-N(1–283) primers (N-F1: 5′-CATACGACGTACCAGATTACGCTTCCACCAGGTCCGTGT-3′), Vim-C(284–466) primers (C-F1: 5′-CATACGTACCAGATTACCTGCAGGAGGCAGAAGAATGG-3′ C-R1: 5′-ACCATGACCGGTGGATCCGCTTCAAGGTCATCGTGATGCTGA-3′), Vim-N283A primer (F1: 5′-TGCCAAGGCTCTGCAGGAGGCAGAAGAATGGT-3′), and R1: 5′-CCTGCAGAGAGCCTTGGCAGCCACACTTTCATATT-3′. PCR amplification was performed using a Novozymes high-fidelity enzyme, recombinase ligated the vector to the different fragments, and the recombination system was placed at 50 °C for 5 min during the reaction, followed by transferring to 4 °C or placing on ice immediately. The recombinant products were clonally transformed into DH-5α, coated with plate-length bacteria, the individual colonies were selected for shaking and amplification, and then the plasmids were extracted and sequenced to verify whether the fragment sequences were successfully constructed.

### 4.9. Mass Spectrometry Analysis

To assess the cleavage of Vim by AEP in vitro, two 10 cm dishes of HEK293 (~1 × 10^7^ cells each dish, obtained from the American Type Culture Collection (ATCC, Innovation, VA, USA) were transfected with 10 μg HA-Vim-EGFP plasmids by the calcium phosphate precipitation method. Then, 48 h after transfection, the cells were collected, washed 3 times in PBS, and lysed in RIPA buffer (20 mM Tris-HCl, pH 7.5, 150 mM NaCl, 1 mM EDTA, 1 mM EGTA, 1% NP-40, 1% sodium deoxycholate, 2.5 mM sodium pyrophosphate, 1 mM β-glycerophosphate, and 1 mM Na_3_VO_4_) with protease inhibitor cocktail on ice for 20 min. The starting material used for this experiment was 1000 μg. The protein concentration was diluted into 5 μg/μL. Samples were precleared by pre-incubation with protein A/G beads for 20 min in 4 °C, then centrifuged for 10 min at 14,000× *g* at 4 °C. The supernatants were incubated with Glutathione Sepharose 4B (10 μL) overnight at 4 °C. After washing with PH = 6.0 RIPA buffer 3 times, the buffer was changed into a PH = 6.0 RIPA buffer, then incubated with 1 μg activated AEP (37 kDa) at 37 °C for 60 min, and shacked every 10 min. The samples were then boiled in 50 μL 1× SDS loading buffer for 10 min and analyzed by Coomassie staining and immunoblotting. Protein samples were in-gel digested with trypsin and analyzed by LC/MS/MS.

### 4.10. Antibodies and Reagents

NeuN (CST, Bossdun, MA, USA; 24307 1:500 For IF), GFAP (CST, 3670, 1:500 for IF), Iba-1 (Abcam, Cambridge, UK; ab178846, 1:500 for IF), Tau-5 (Invitrogen, Carlsbad, CA, USA; AHB0042, 1:500 for IF), AT8 (Invitrogen, MN1020, 1:500 for IF), legumain (D6S4H) rabbit mAb (AEP, anti-δ-secretase) (CST, 93627S, 1:1000 For WB, 1:500 for IF), actin (Servicebio, Wuhan, China; GB15001-100, 1:1000 for WB), GST (Proteintech, Wuhan, China; 10000-0-AP, 1:1000 for WB), EGFP (Beyotime, Shanghai, China; AG281, 1:1000 for WB, 1:500 for IF), rabbit IgG isotype control (Zenbio, Chengdu, China; A00002), mouse IgG isotype control (Zenbio, A00001), purified anti-β amyloid, 14-24 antibody (4G8) (Biolegend, San Diego, CA, USA; 800702, 1:1000 for WB, 1:500 for IF), APOE (Zenbio, R381129, 1:1000 for WB, 1:500 for IF), MAO-B (Abclonal, Wuhan, China; A1568, 1:1000 for WB, 1:500 for IF), anti-vimentin (Abclonal, A1999607, 1:1000 for WB, 1:500 for IF).

### 4.11. Statistical Analysis

All data are presented as averaged soil SEMs from three or more independent experiments and illustrated with GraphPad Prism (version 8.0). One-way ANOVA was applied to confirm significant principal effects and differences between three or more groups, followed by Tukey or least significant difference (LSD) multiple comparisons for post hoc testing. For time-course studies, significance levels were determined using two-way ANOVA and Bonferroni multiple comparisons. Kruskal–Wallis and Dunn multiple comparisons were used to analyze the between-group ratios. The *p* value of <0.05 was considered statistically significant.

## Data Availability

The data that support the findings of this study are available from the corresponding author upon reasonable request.

## Supplementary Materials

The following supporting information can be downloaded at: https://www.mdpi.com/article/10.3390/ijms26072857/s1.

## Abbreviations

The following abbreviations are used in this manuscript:

AD	Alzheimer’s disease
EOAD	Early onset Alzheimer’s disease
PD	Parkinson’s disease
sCJD	Creutzfeldt–Jakob disease
Vim	Intermediate filament protein vimentin
Aβ	Amyloid-β
AEP	Asparagine endopeptidase
GFAP	Glial fibrillary acidic protein
MAO	Monoamine oxidase
ApoE	Apolipoprotein E
LDLR	Low-density lipoprotein receptor
