# Supplementary material for: Vimentin Fragmentation and Its Role in Amyloid-Beta Plaque Deposition in Alzheimer’s Disease
Source: Int J Mol Sci. 2025 Mar 21;26(7):2857. doi: 10.3390/ijms26072857 (PMC11988971; doi:10.3390/ijms26072857)
Supplement: Supplementary file 1 [file ijms-26-02857-s001.zip › ijms-3531710-supplementary.pdf]

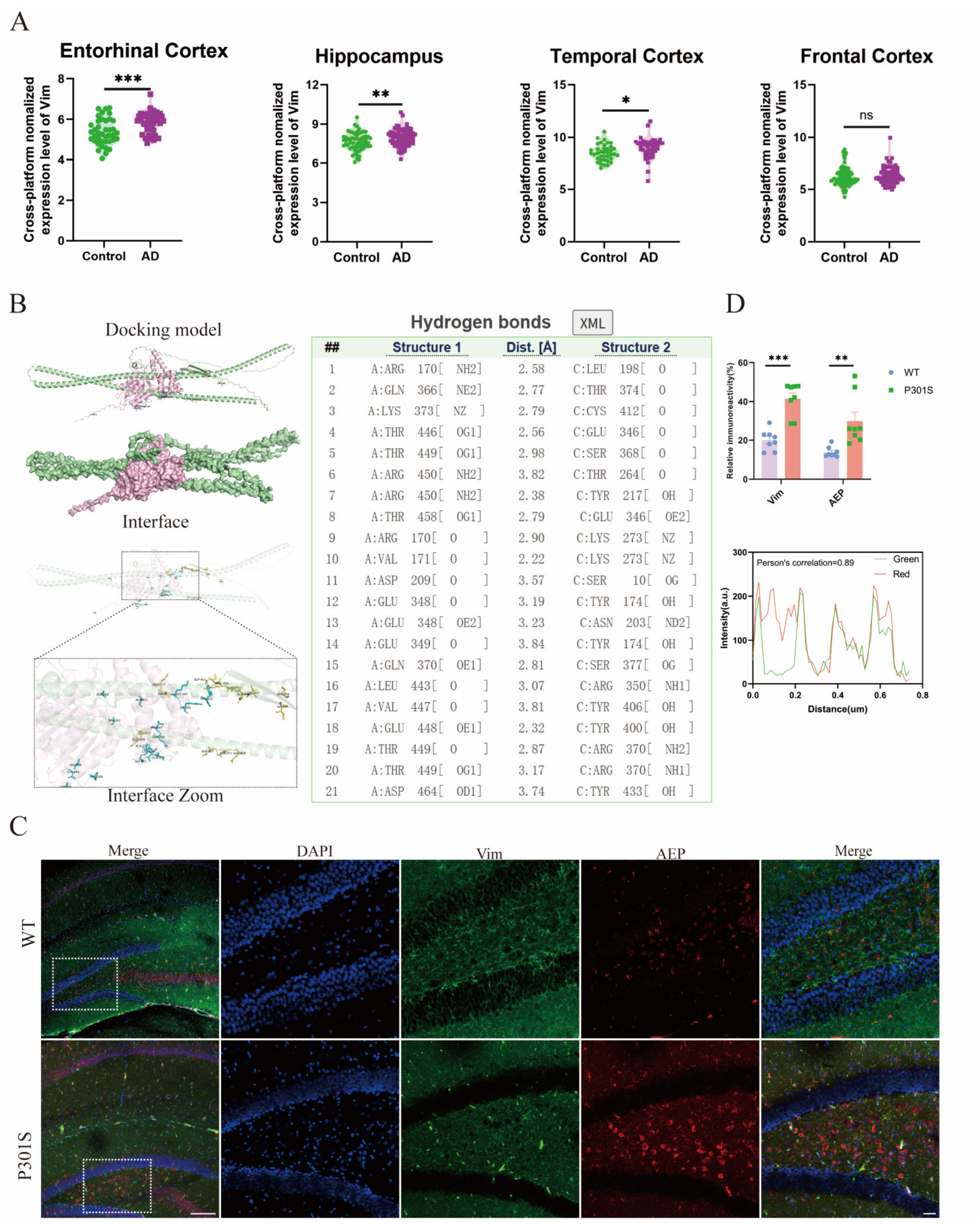

Supplementary Figure S1: Vim mRNA is upregulated in multiple AD brain regions. AEP does not interact with Vim in DG region of P301S mice.

(A) The expression of Vim in different brain regions. Data were obtained from AlzDate and GEO databases (Dataset of Entorhinal Cortex: GSE26927, GSE26972, GSE48350, GSE5281.Dataset of Hippocampus: GSE28146, GSE29378, GSE36980, GSE48350, GSE5281.Dataset of Temporal Cortex: GSE29652, GSE36980, GSE37263, GSE5281.Dataset of Frontal Cortex: GSE12685, GSE36980, GSE48350, GSE5281,

GSE53890, GSE66333.), and R software and RStudio were used for data statistics. **(B)** The structures of Vim and AEP proteins were obtained from the UniProt database. Molecular docking was performed using GRAMM, and the results were processed through EMBL-EBI. The potential interactions between Vim and AEP were visualized using PyMOL. In the visualization, Vim is shown in yellow and AEP in blue, with the purple lines indicating the interactions between the binding sites. Specifically, the interaction sites on the Vim structure include ASP-367, ASN-371, and GLU-374, while those on the AEP structure include GLY-375, GLU-413, LYS-347, LYS-351, and GLY-378. Additionally, AEP-TYP-433 interacts with Vim-ASP-451. **(C)** Immunohistochemistry reveals the expression patterns of AEP and Vim in brain sections from P301S and WT mice. Scale bar, 20 μm. **(D)** Quantitative and co-localization analysis. Data are mean ± SEM; $n \geq 3$; *$p < 0.05$, **$p < 0.01$, ***$p < 0.001$, by one-way ANOVA.

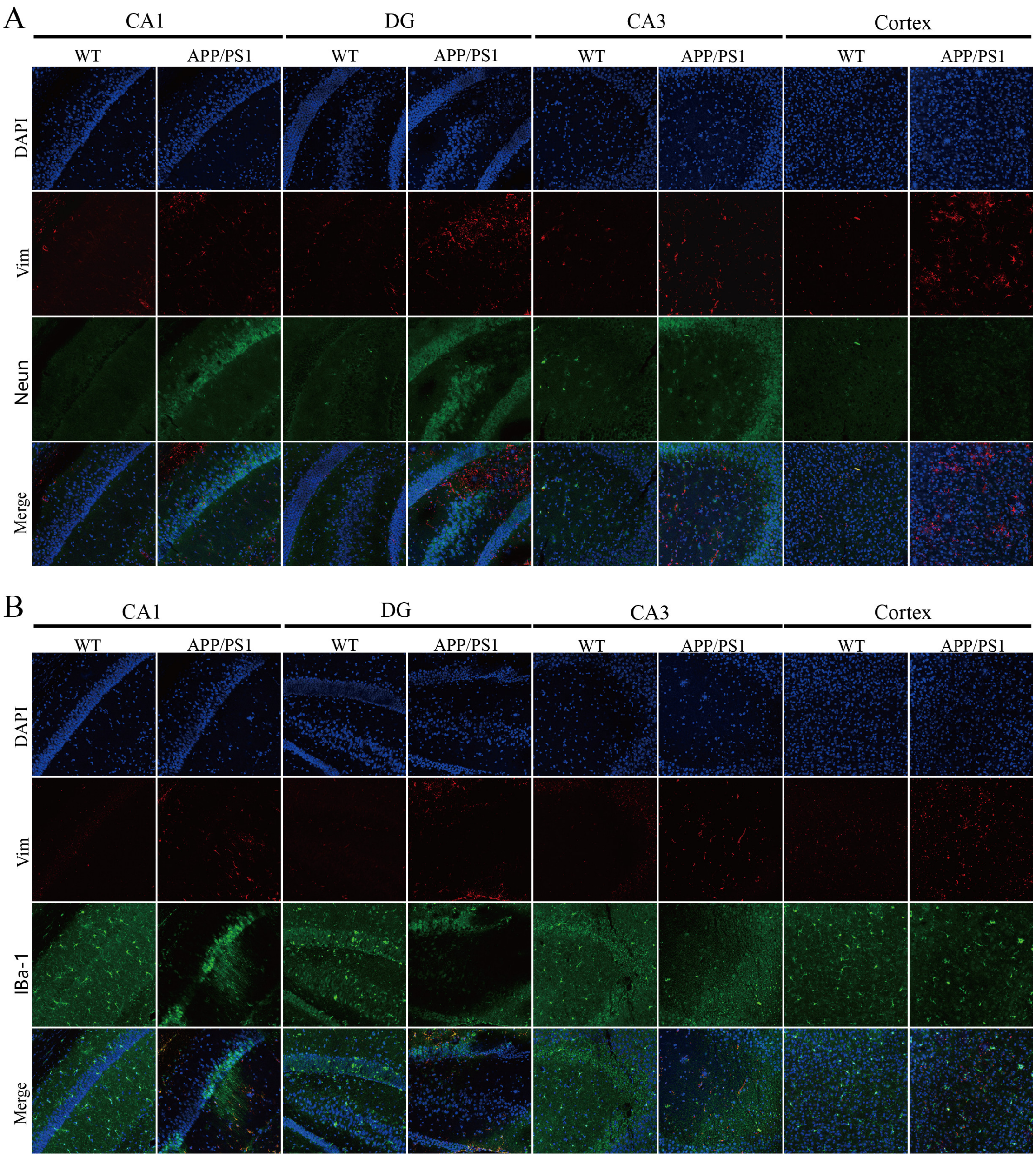

Supplementary Figure S2: Vim expression in different neural cells of APP/PS1 mice.

(A) Immunohistochemistry demonstrated that Vim partially colocalized with Neun in various brain regions, including the CA1, CA3, DG, and cortex, in both APP/PS1 and WT mice. Scale bar, 40 µm.**(B)** Immunohistochemistry demonstrated that Vim partially colocalized with Iba-1 in various brain regions, including the CA1, CA3, DG, and cortex, in both APP/PS1 and WT mice. Scale bar, 40 µm.

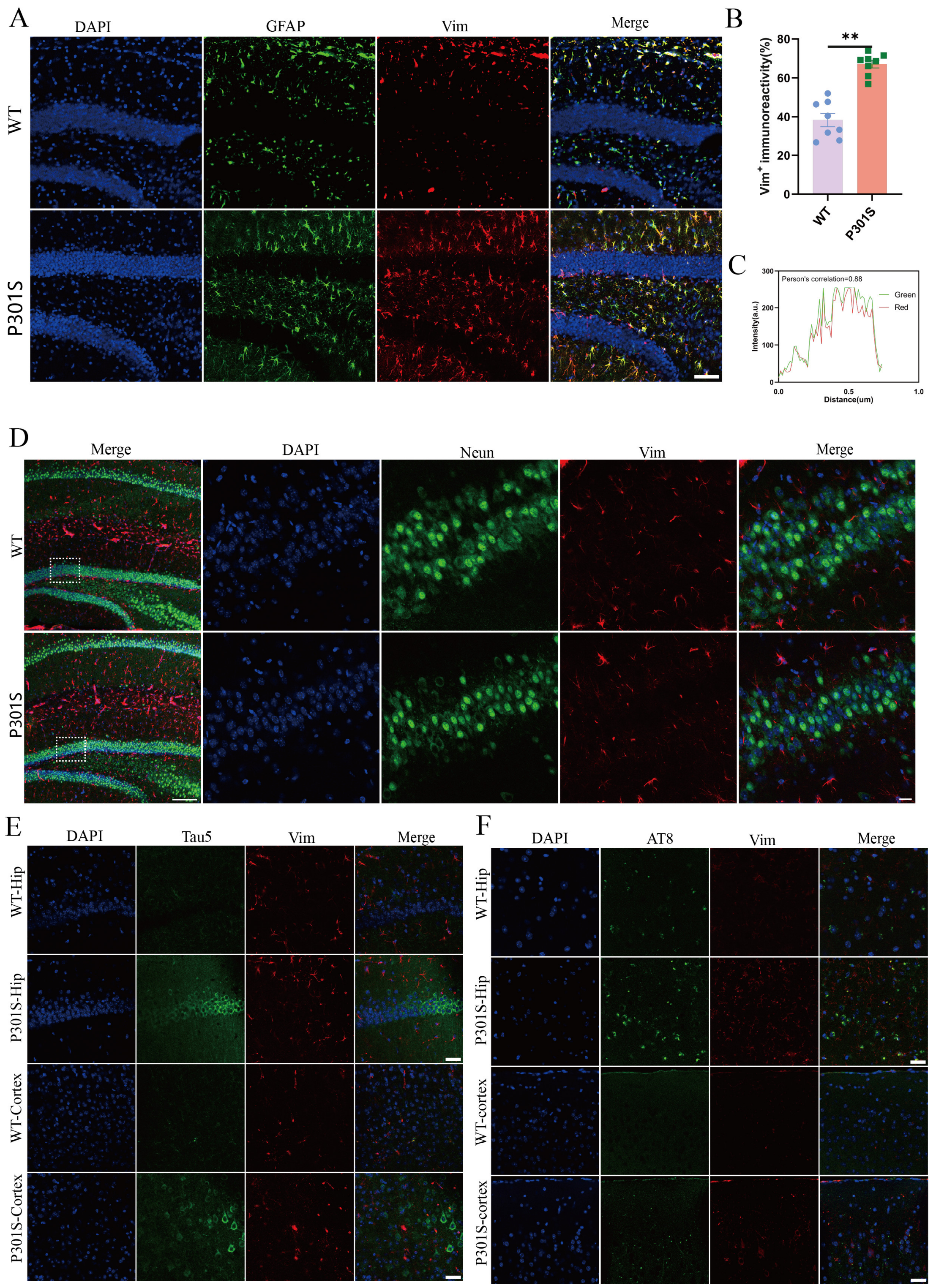

Supplementary Figure S3: The relationship between Vim and Tau pathology.

(A-C) Immunohistochemistry revealed that Vim colocalized with GFAP in the hippocampus of both P301S and wild-type (WT) mice. Scale bar, 20μm. Quantitative statistical analysis protein level. Data are mean ± SEM; $n \geq 3$; $^{**}p < 0.01$ by one-way ANOVA. **(D)** Immunohistochemistry demonstrated that seldom Vim colocalized with

Neun in the hippocampus of both P301S and wild-type (WT) mice. Scale bar, 20μm. **(E)** Immunohistochemistry revealed that the little Vim colocalized with Tau5 in the hippocampus of both P301S and wild-type (WT) mice. Scale bar, 40μm. **(F)** Immunohistochemistry revealed that the little Vim colocalized with AT8 in the hippocampus of both P301S and wild-type (WT) mice. Scale bar, 40μm.

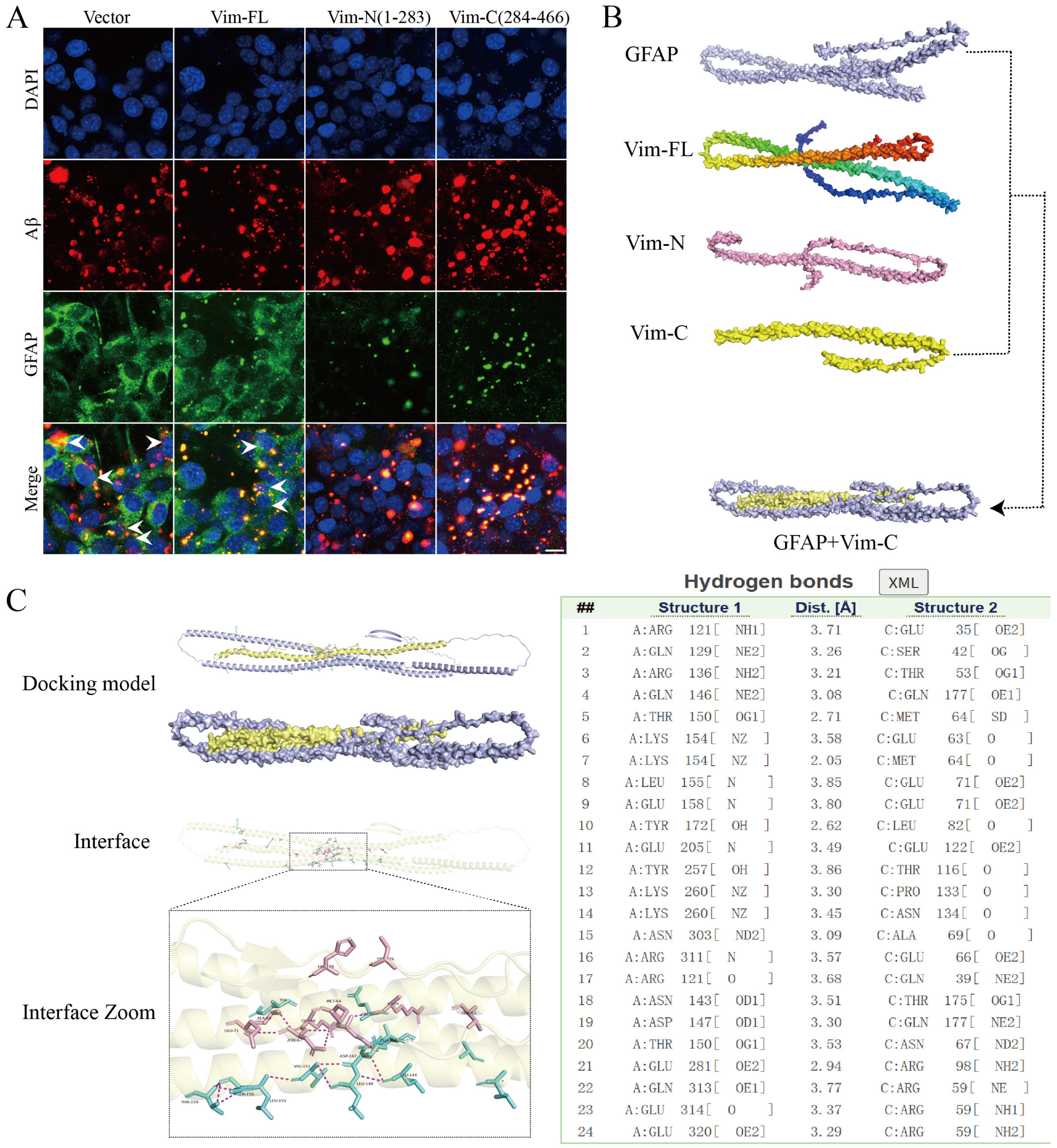

##	Structure 1	Dist. [Å]	Structure 2
1	A:ARG 121[NH1]	3.71	C:GLU 35[OE2]
2	A:GLN 129[NE2]	3.26	C:SER 42[OG]
3	A:ARG 136[NH2]	3.21	C:THR 53[OG1]
4	A:GLN 146[NE2]	3.08	C:GLN 177[OE1]
5	A:THR 150[OG1]	2.71	C:MET 64[SD]
6	A:LYS 154[NZ]	3.58	C:GLU 63[O]
7	A:LYS 154[NZ]	2.05	C:MET 64[O]
8	A:LEU 155[N]	3.85	C:GLU 71[OE2]
9	A:GLU 158[N]	3.80	C:GLU 71[OE2]
10	A:TYR 172[OH]	2.62	C:LEU 82[O]
11	A:GLU 205[N]	3.49	C:GLU 122[OE2]
12	A:TYR 257[OH]	3.86	C:THR 116[O]
13	A:LYS 260[NZ]	3.30	C:PRO 133[O]
14	A:LYS 260[NZ]	3.45	C:ASN 134[O]
15	A:ASN 303[ND2]	3.09	C:ALA 69[O]
16	A:ARG 311[N]	3.57	C:GLU 66[OE2]
17	A:ARG 121[O]	3.68	C:GLN 39[NE2]
18	A:ASN 143[OD1]	3.51	C:THR 175[OG1]
19	A:ASP 147[OD1]	3.30	C:GLN 177[NE2]
20	A:THR 150[OG1]	3.53	C:ASN 67[ND2]
21	A:GLU 281[OE2]	2.94	C:ARG 98[NH2]
22	A:GLN 313[OE1]	3.77	C:ARG 59[NE]
23	A:GLU 314[O]	3.37	C:ARG 59[NH1]
24	A:GLU 320[OE2]	3.29	C:ARG 59[NH2]

Supplementary Figure S4: Vim interacts with GFAP

(A) Different fragments of Vim were transfected into SW1783 cells, and the cells were fixed after 24h of exogenous Aβ-594 treatment, and the phagocytosis of exogenous Aβ was detected by immunofluorescence staining. **(B)** Schematic diagram of the docking

of GFAP with Vim molecule. **(C)** The structures of Vim-C and GFAP were obtained from the UniProt database. Molecular docking was performed using GRAMM, and the results were processed through EMBL-EBI. The potential interactions between Vim-C and GFAP were visualized using PyMOL. In the visualization, Vim-C is shown in red and GFAP in green, with the purple lines indicating the interactions between the binding sites. Specifically, the interaction sites between Vim-C and GFAP on the right figure.

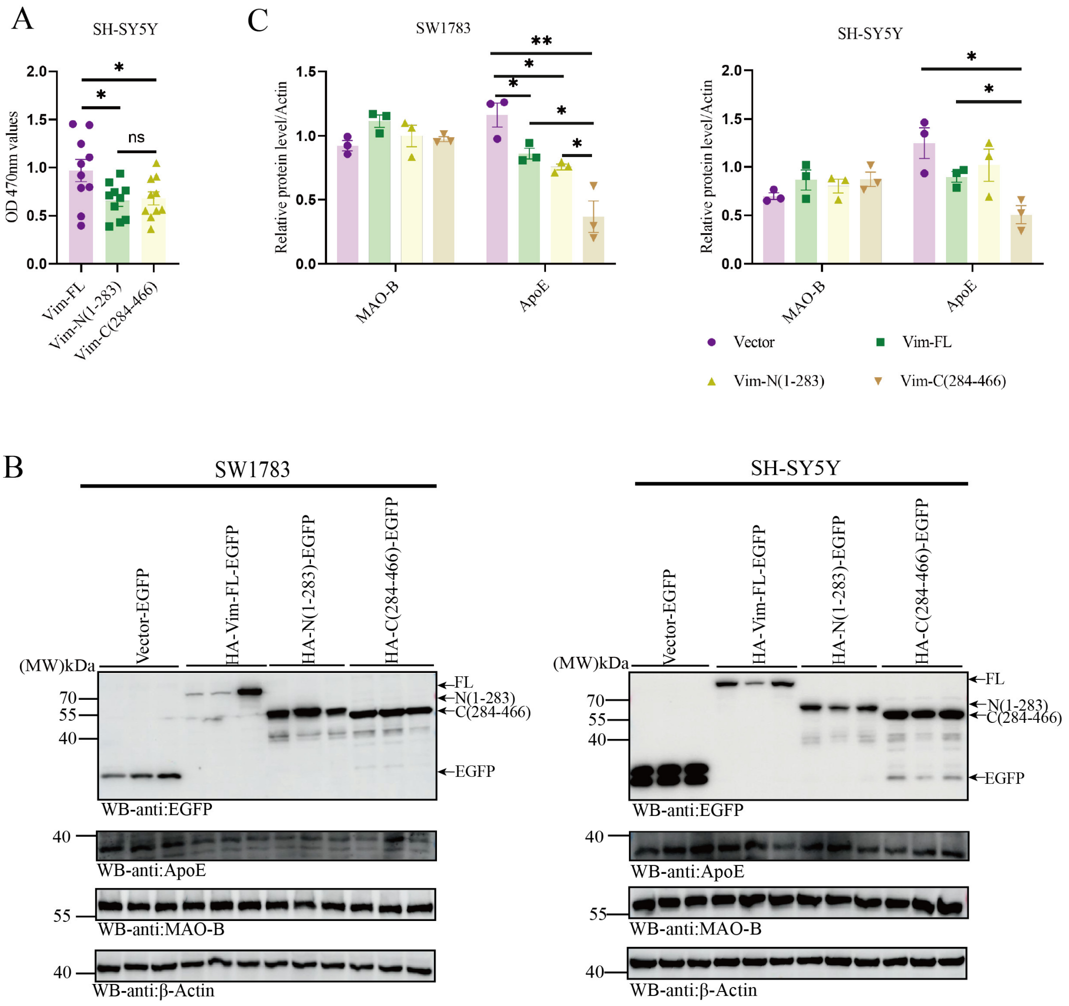

Supplementary Figure S5: Vim fragmentations impact ApoE but not MAO-B expression.

(A) The MTT assay was employed to assess the viability of SH-SY5Y cells transfected with Vim-FL (full-length Vim), Vim-N (N-terminal fragment), Vim-C (C-terminal fragment), and Vector control. The results indicated that Vim fragmentation significantly increased cell death in SH-SY5Y cells. Data are mean ± SEM; $n \geq 3$; $*p <$

0.05 by one-way ANOVA. **(B)** Western blot analysis of ApoE and MAO-B protein level with different Vim fragmentations in SW1783 and SH-SY5Y cell. **(C)** Quantitative statistical analysis of protein level. Data are mean ± SEM; $n \geq 3$; $*p < 0.05$, $**p < 0.01$by one-way ANOVA.

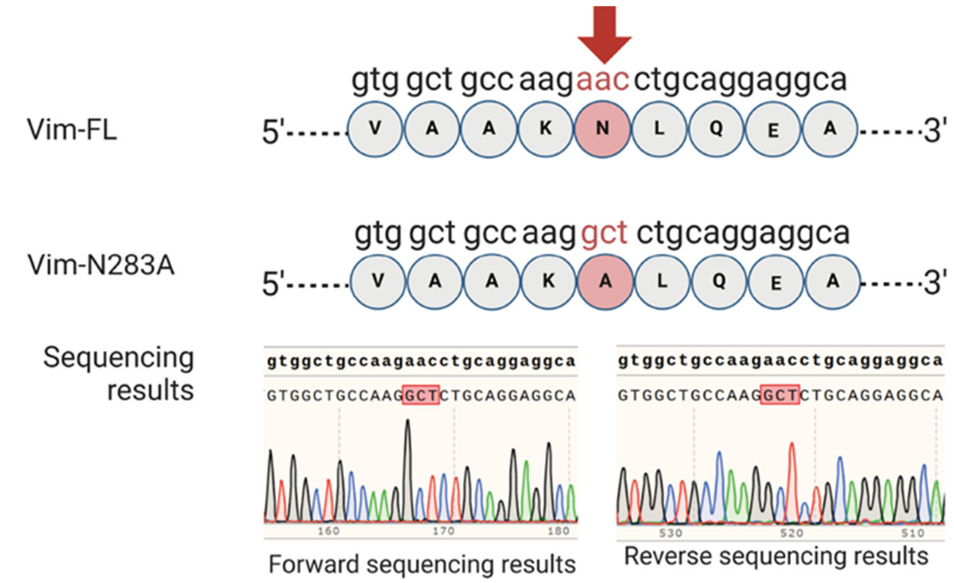

Supplementary Figure S6: Schematic diagram and sequencing results of the Vim-N283A mutant plasmid construction.

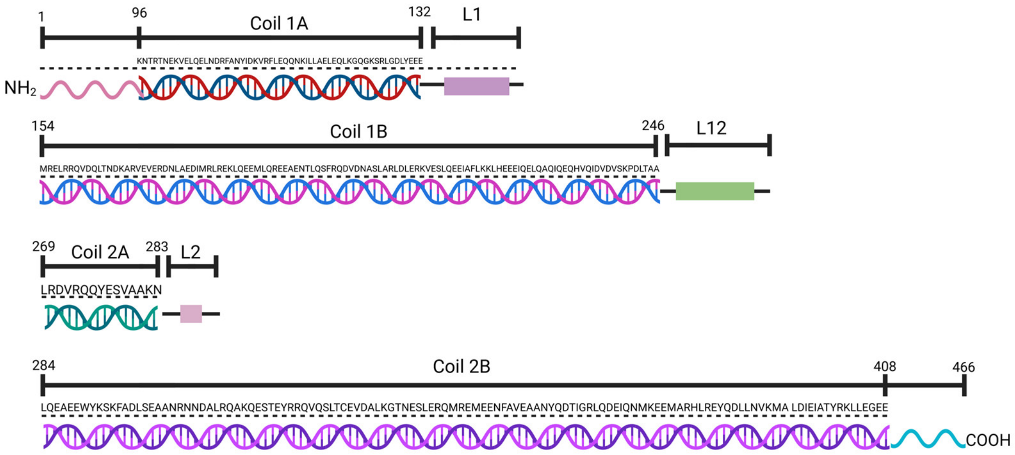

Supplementary Figure S7: Diagram of Vimentin protein structure.
The Vim protein mainly includes two main bodies, Coil A and Coil B, which are connected by L2 structure, among which Coil B is the part of Vim-C terminal found in this study.
